# Supplementary material for: Genomic and Epigenetic Changes Drive Aberrant Skeletal Muscle Differentiation in Rhabdomyosarcoma
Source: Cancers (Basel). 2023 May 18;15(10):2823. doi: 10.3390/cancers15102823 (PMC10216238; doi:10.3390/cancers15102823)
Supplement: Supplementary file 1 [file cancers-15-02823-s001.zip › cancers-2323056-supplementary.pdf]

Table S1. Drugs inducing myogenic differentiation in RMS cells in preclinical studies, which have been examined in early phase clinical trials in children and adolescents.

Drug	Cellular target	Mechanism of action	Clinical trial	R2P2	RMS	Possible partner drugs
**Trametinib (1)**	MEK1/2	MAPK pathway	NCT02124772 (completed)	0,032 mg/kg PO once daily (< 6yo); 0,025 mg/kg PO once daily (>/= 6yo)	n/a	+ RAF-I, + multi-TKI, + IGFR1-I-i
**Erlotinib (2)**	EGFR	EGFR pathway	NCT00418327 (completed)	125 mg/m^2 PO once daily	not included	+ irinotecan & vincristine
**Tazemetostat (3)**	EZH2	PRC2	NCT02601937 (completed)	520 mg/ m^2 PO once daily	not included	
**Vorinostat (4)**	pan-HDAC	histone acetylation	NCT01422499 (completed)	230 mg/m^2 PO once daily	RMS included (n=3; no response)[1]	
**Vorinostat (5)**	pan-HDAC	histone acetylation	NCT01422499 (completed)	n/a	STS included (n=1; no response)[1]	
**Entinostat (6)**	HDAC1/3	histone acetylation	NCT02780804 (completed)	4 mg/m^2 IV once weekly	RMS included (n=1; no response)[1]	
**Cixutumumab (7)**	IGF1R	IGF1R	NCT00831844 (completed)	9 mg/kg IV weekly	RMS included (n=20; 1 PR)[2]	+ trametinib
**Ganitumab (combined w/ dasatinab) (8)**	IGF1R	IGF1R	NCT0304170 (terminated)	Ganitumab 18 mg/kg IV every 2 weeks, (dasatinib 60 mg/m^2 PO once daily)	RMS included (n=9; 1 PR/ 1 SR)	
**Panobinostat**	pan-HDAC	histone acetylation	NCT02717455 (active, not recruiting)	n/a	not included	
**Cabozantinib (9)**	MET	P3F downstream signaling	NCT02867592 (active, not recruiting)	40 mg/m^2 PO once daily	included [3]	
**AZD1775 (combined w/ irinotecan)** [4]	WEE1	Cell cycle	NCT02095132 (active, not recruiting)	AZD1775 85 mg/m^2 PO days 1-5, irinotecan 90 mg/m^2 PO days 1-5	included [3]	+ vincristine

**BMS-986158**	BRD4	RMS CRC	NCT03936465 (active, recruiting)	n/a	n/a	+ CDK9-i
**Erdafitinib**	pan-FGFR	FGFR4 (P3F downstream signaling)	NCT04083976 NCT03155620 (both active, recruiting)	n/a	n/a	
**Ponatinib**	Pan-FGFR	FGFR4 (P3F downstream signaling)	NCT03934372 (active, recruiting)	n/a	n/a	

[1] no objective responses in RMS patients observed; [2] ARST043: addition to intensive multi-agent chemotherapy in patients with metastatic RMS feasible; but no clinical benefit; [3] no data on objective responses in patients with RMS available yet; [4] vincristine not administered despite preclinical evidence indicating that vincristine significantly enhanced anti-RMS efficacy of AZD1775 and irinotecan in mice

Abbreviations: R2P2, recommended phase 2 dose; RMS, rhabdomyosarcoma; PO, per os; IV, intravenously; yo, years old; STS, soft-tissue sarcoma; TKI, tyrosine kinase inhibitor; PR, partial response; SD, stable disease.

References:

1. Bouffet E, Geoerger B, Moertel C, Whitlock JA, Aerts I, Hargrave D*, et al.* Efficacy and Safety of Trametinib Monotherapy or in Combination With Dabrafenib in Pediatric BRAF V600-Mutant Low-Grade Glioma. J Clin Oncol **2023**;41:664-74
2. Geoerger B, Hargrave D, Thomas F, Ndiaye A, Frappaz D, Andreiuolo F*, et al.* Innovative Therapies for Children with Cancer pediatric phase I study of erlotinib in brainstem glioma and relapsing/refractory brain tumors. Neuro Oncol **2011**;13:109-18
3. Chi SN, Bourdeaut F, Laetsch TW, Fouladi M, Macy ME, Makin G*, et al.* Phase I study of tazemetostat, an enhancer of zeste homolog-2 inhibitor, in pediatric pts with relapsed/refractory integrase interactor 1-negative tumors. JCO **2020**;ASCO Annual Meeting/ asbtract
4. Fouladi M, Park JR, Stewart CF, Gilbertson RJ, Schaiquevich P, Sun J*, et al.* Pediatric phase I trial and pharmacokinetic study of vorinostat: a Children's Oncology Group phase I consortium report. J Clin Oncol **2010**;28:3623-9
5. van Tilburg CM, Milde T, Witt R, Ecker J, Hielscher T, Seitz A*, et al.* Phase I/II intra-patient dose escalation study of vorinostat in children with relapsed solid tumor, lymphoma, or leukemia. Clin Epigenetics **2019**;11:188
6. Bukowinski A, Chang B, Reid JM, Liu X, Minard CG, Trepel JB*, et al.* A phase 1 study of entinostat in children and adolescents with recurrent or refractory solid tumors, including CNS tumors: Trial ADVL1513, Pediatric Early Phase-Clinical Trial Network (PEP-CTN). Pediatr Blood Cancer **2021**;68:e28892
7. Weigel B, Malempati S, Reid JM, Voss SD, Cho SY, Chen HX*, et al.* Phase 2 trial of cixutumumab in children, adolescents, and young adults with refractory solid tumors: a report from the Children's Oncology Group. Pediatr Blood Cancer **2014**;61:452-6
8. Akshintala S, Bernstein D, Glod J, Kaplan RN, Shern JF, Yohe ME*, et al.* Results of a phase I trial of ganitumab plus dasatinib in patients with rhabdomyosarcoma (RMS). JCO **2022**;ASCO Annual Meeting/ abstract
9. Akshintala S, Widemann BC, Barkauskas DA, Hall D, Reid JM, Voss SD*, et al.* Phase 2 trial of cabozantinib in children and young adults with refractory sarcomas, Wilms tumor, and rare tumors: Children's Oncology Group Study (ADVL1622). . ASCO Annual Meeting/ abstract **2021**
